# Complete genome sequences of *Vibrio* species, degraders of sulfated polysaccharide ulvan extracted from a green algae *Ulva ohnoi*

**DOI:** 10.1128/mra.00113-26

**Published:** 2026-03-19

**Authors:** Nur Athiqah Zhafirah, Masanori Hiraoka, Miho Satoh, Mukrimin Mukrimin, Syamsuddin Millang, Kouhei Ohnishi

**Affiliations:** 1 Faculty of Forestry, Hasanuddin University 64739, Makassar, South Sulawesi, Indonesia; 2 Usa Marine Biological Institute, Kochi University, Tosa, Kochi, Japan; 3 Institute of Molecular Genetics, Kochi University, Nankoku, Kochi, Japan; Montana State University, Bozeman, Montana, USA

**Keywords:** ulvan, marine microbiology, utilization

## Abstract

We present the complete genome sequences of *Vibrio* sp. strains that degrade algal sulfated polysaccharide, ulvan. Polysaccharide utilization loci (PULs) were found in all ulvan-utilizing strains. A comparison of PULs between *Alteromonas* and *Vibrio* will elucidate the ulvan degradation pathway.

## ANNOUNCEMENT

The green algae *Ulva ohnoi* has a high growth rate among *Ulva* species (1), often leading to green tides in coastal sea areas (2). *U. ohnoi* contains approximately 35 wt % ulvan (3), a complex water-soluble sulfated polysaccharide mainly composed of L-rhamnose-3-sulfate, glucuronic acid, and iduronic acid. Ulvan polysaccharide and depolymerized oligoulvan can be used in various food, feed, biomedical, paint, and textile industries (4). Ulvan-utilizing bacteria contain genes involved in ulvan degradation in a gene cluster called the polysaccharide utilization locus (PUL) (5).

*U. ohnoi* cultivated at *onshore* aquaculture facilities was enclosed in a mesh net and deployed for 1 week in August 2022 at 33.43°N, 133.42°E in Uranouchi Bay (Kochi, Japan) as a bait substrate to enrich and capture ulvan-degrading bacteria. The recovered green algae were placed in a 250-mL flask with 40 mL of autoclaved artificial seawater (Tetra Marin Salt Pro, Tetra Japan) containing 0.2% (wt/vol) *U. ohnoi* ulvan as the sole carbon source and incubated at 25°C for 2 days. Enriched cells were spread on artificial seawater agar medium with 0.2% (wt/vol) ulvan as the sole carbon source and incubated at 25°C for two days. The isolated bacteria were grown again on the same media, and three colonies were selected. The V1–V3 regions of 16S rDNA were amplified from the strains using the primers proR2 (5′-AGAGTTTGATCMTGGCTCAG-3′) and 534R (5′-ATTACCGCGGCTGCTGG-3′). The nucleotide sequences of the amplified fragments were determined using a 3130 genetic analyzer (Applied Biosystems). All three strains belonged to the genus *Vibrio*.

Genomic DNA was extracted from cells grown overnight in Marine Broth 2216 (Sigma-Aldrich) at 25°C using the Wizard HMW DNA Extraction kit (Promega). Purified DNA (78, 79, and 126 ng/μL for KUL70, KUL78, and KUL80, respectively; QuantiFluor ONE dsDNA dye, Promega) was used for short- and long-read sequencing. For short-read sequencing, genomic DNA was fragmented to 150–350 bp, and libraries were built using the NEBNext Ultra II FS DNA Library Prep Kit for Illumina (NEB) with NEBNext Multiplex Oligos for Illumina. The pooled libraries (at a concentration of 20 pM) were sequenced on an Illumina MiSeq (MiSeq Reagent kit v3, 150 cycles), generating 75-bp paired-end reads. Adapter-trimmed reads with Phred-encoded quality scores > 30 were selected using MiSeq Reporter 2.6.2.

For long-read sequencing, genomic DNA was processed using a 1D Ligation Sequencing kit (SQK-LSK109; Oxford Nanopore Technologies) with Native Barcoding Expansion (EXP-NBD104). The pooled libraries were sequenced without size selection on MinION with Flow Cell (R9.4.1). Basecalling was performed in real-time using MinKNOW version 22.05.5 with an integrated Dorado basecaller. The fast basecalling model for R9.4.1 flow cells (dna_r9.4.1_450bps_fast) was used. Basecalling was performed on a GPU with default parameters. Quality scores were measured on a logarithmic Phred scale. Reads with a quality score below a minimum of 8 were removed as failed reads.

The hybrid assembly with long and short reads was conducted using an assembly pipeline for bacterial genomes, Trycycler v0.5.4 (6). Trycycler uses TBLASTN to search for the *dnaA* allele in each completed replicon, and the sequence is circularized (7). Genome completeness and contamination were evaluated using CheckM v1.0.18 (8) with a lineage-specific workflow (lineage_wf). All genome assemblies were phylogenetically placed within the genus *Vibrio*, and the quality metrics are shown in Table 1. Gene annotation was conducted using DFAST prokaryotic genome annotation pipeline v.1.6.0 (9). Default parameters were used for all software, unless otherwise specified.

**TABLE 1:** Characterization of three *Vibrio* strains

Strain	Accession no.	Genome size (bp)	Completeness; contamination (%)	GC content (%)	No. of CDSs, rRNAs, and tRNAs	Bioproject; biosample	Sequencing	DRA accession no.	Coverage (×)	Number of reads of MiSeq	Mean read length and quality; median read length and quality; number of reads; read length N50; total bases of MinION
KUL70	AP045834	4,799,380	99.74	44.9	4,095	PRJDB35666	MiSeq	DRX686772	45	1,411,859	4,124.6, 10.0,
	AP045835 AP045836		0.98		40 132	SAMD01598106	MinION	DRX686769	120		2,811.0, 10.1, 140,062, 5,381, and 577,705,068
KUL78	AP045837	5,989,974	98.82	46.0	5,510	PRJDB35666	MiSeq	DRX686773	13	517,147	4,204.8, 10.0,
	AP045838 AP045839 AP045840		3.76		31 112	SAMD01598107	MinION	DRX686770	41		2,791.0, 10.0, 57,996, 5,383, and 243,862,671
KUL80	AP045841	5,712,820	99.12	46.1	5119	PRJDB35666	MiSeq	DRX686774	35	1,331,052	7,028.6, 10.0,
	AP045842 AP045843		3.06		34 117	SAMD01598108	MinION	DRX686771	41		3,580.0, 10.0, 32,925, 13,011, and 231,417,115

The characteristics of the sequenced genomes are listed in Table 1. In contrast to *Alteromonas* species (10), which contain a long-type PL24 family ulvan lyase (*ullA*), short-type PL25 ulvan lyase (*ullB*)*,* and PL25 ulvan lyase (*ullC*) in the PUL, these strains contain only *ullB* and *ullC* genes.

## Data Availability

This whole-genome shotgun (WGS) project was deposited in the DDBJ/EMBL/GenBank. The versions described in this paper are the first. The read archives were deposited in the DDBJ DRA. WGS and DRA accession numbers are listed in Table 1.
